# Feasibility of pulsed field ablation for atrial fibrillation under mild conscious sedation

**DOI:** 10.1007/s10840-024-01961-1

**Published:** 2024-12-02

**Authors:** Peter Calvert, Mark T. Mills, Ben Murray, Jonathan Kendall, Justin Ratnasingham, Vishal Luther, Dhiraj Gupta

**Affiliations:** 1 Liverpool Centre for Cardiovascular Science at University of Liverpool, Liverpool John Moores University and Liverpool Heart & Chest Hospital, Liverpool, UK; 2 Liverpool Heart & Chest Hospital NHS Foundation Trust, Thomas Drive, Liverpool, L14 3PE UK; 3 Department of Cardiology, Liverpool Heart & Chest Hospital, Thomas Drive, Liverpool, L14 3PE UK

**Keywords:** Atrial fibrillation, Catheter ablation, Pulsed field ablation, Sedation

## Abstract

**Background:**

Pulsed field ablation (PFA) is a new modality for pulmonary vein isolation (PVI) for atrial fibrillation (AF). PFA is performed under general anaesthetic (GA) or deep sedation with propofol, but this requires anaesthetic support in many countries, restricting use. No study has tested the feasibility of PFA under mild conscious sedation (MCS).

**Methods:**

We prospectively recruited patients undergoing PFA PVI, offered the option of MCS delivered by electrophysiologists, and compared these with patients who opted for GA. MCS comprised intravenous midazolam and fentanyl. All procedures were performed under anaesthetic supervision in case of requirement to convert to GA, which formed the primary outcome.

**Results:**

Twenty-three patients were recruited (8 MCS, 15 GA). One patient (1/8 [12.5%]) required conversion from MCS to GA. Total procedural times were similar between groups (MCS 92 ± 12.4 min vs. GA 101 ± 17.3 min; *p* = 0.199). High mean sedative doses were required in the MCS group (5.12 ± 0.83 mg midazolam and 209 ± 40 mcg fentanyl). Median intraprocedural pain perception by the patient, rated from 0 to 100 was 45 (IQR 22.5–72.5) in the MCS group. Post-procedural groin pain (0 [0–0] vs. 5 [0–35]; *p* = 0.027) and throat pain (0 [0–0] vs. 10 [5–40]; *p* = 0.001) were lower in the MCS group.

**Conclusion:**

PFA under MCS is feasible in selected patients but pain and tolerance may be suboptimal, and high sedative doses are required.

**Graphical abstract:**

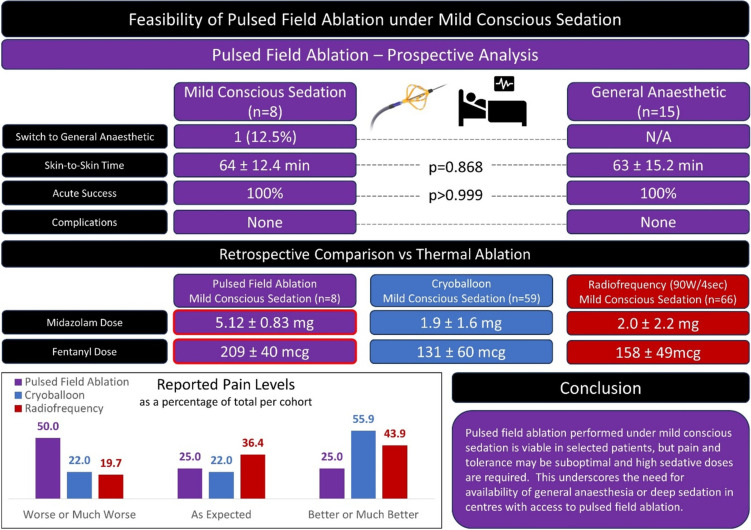

**Supplementary Information:**

The online version contains supplementary material available at 10.1007/s10840-024-01961-1.

## Introduction

Pulsed field ablation (PFA) is a newly emerging ablation modality for achieving pulmonary vein isolation (PVI) in patients with atrial fibrillation (AF). In existing studies, PFA has demonstrated comparable safety and effectiveness to traditional thermal ablation modalities [1, 2].

The FaraPulse system (Boston Scientific) is the first commercially available single-shot PVI device, delivering PFA over a large footprint. The catheter is advanced over a guidewire into each pulmonary vein, after which typically 4 pulses are delivered in ‘basket’ configuration and 4 in ‘flower’ configuration. Whilst PFA is considerably quicker than thermal ablation, a significant disadvantage is its reliance upon general anaesthesia (GA), due to the perception that the electrical impulses used to damage myocytes result in potentially painful skeletal muscle contraction. Although some centres, mainly in Europe, perform PFA under deep sedation using propofol infusion [3], this approach is not possible without anaesthetic supervision in the majority of centres worldwide [4]. Therefore, many centres across the world are unable to offer a full PFA service due to limited access to anaesthetic support.

Mild conscious sedation (MCS) with a combination of benzodiazepines and opiates is routinely used for thermal ablation in the United Kingdom (UK) and other countries [5, 6]. No study has previously tested the feasibility of PFA under MCS. We therefore designed a study to test this approach as a potential way to maximise the benefits of PFA without requiring anaesthetic oversight.

## Methods

### Study design

This study was a single-centre, prospective, non-randomised feasibility study of PFA under MCS compared against PFA under GA. The study was approved by our local Research & Innovation Committee and by the National Research Ethics Committee (IRAS 320883). The study was registered on Clinicaltrials.gov prior to commencement (NCT06014814). All patients provided informed, written consent for their ablation procedures and for study involvement. As a feasibility study, no power calculation was undertaken, but our recruitment target was arbitrarily set at 20 patients per arm.

Patients listed for PFA PVI were invited to consider whether they would prefer MCS rather than GA after an informed discussion about the potential pros and cons of this approach. The primary benefits of MCS were expected to be quicker recovery and less throat discomfort from intubation, with possibly easier same-day discharge. The main downside was likely to be pain or discomfort caused by the ablation itself. The patients were educated about the procedure workflow and the transient nature of the potentially painful PFA pulse applications, namely, ≥ 8 applications per PV, each lasting 2.5 s.

The decision of allocation to either arm was primarily down to patient preference, as long as there were no contraindications (see inclusion and exclusion criteria below). We intentionally did not undertake randomisation as we recognised that this may induce selection bias—i.e. patients who wanted GA but were randomised to MCS may simply withdraw from the study. Our goal was to analyse tolerance in the most optimal and motivated patient group possible, with the view that if even these patients could not tolerate PFA under MCS, it would be highly unlikely to work in a randomised setting.

### Inclusion and exclusion criteria

Given the feasibility nature of this study, minimal inclusion and exclusion criteria were applied. Patients who were undergoing catheter ablation for AF using PFA were approached for inclusion. Patients who were considered high risk for MCS (BMI ≥ 40, obstructive sleep apnoea, or other airway concerns) were only considered eligible for the GA arm. Similarly, patients in whom posterior wall isolation was planned were not considered for MCS due to the more extensive nature of this ablation.

### Ablation procedures and sedation technique

All ablation procedures were performed on GA lists in case conversion from MCS to GA was required for non-tolerance. Patients in the GA arm underwent routine induction of anaesthesia at the discretion of the anaesthetist. Induction of anaesthesia was achieved with a propofol bolus of 1–2 mg/kg and a muscle relaxant of the anaesthetist’s choice to facilitate endotracheal intubation, followed by maintenance of anaesthesia using sevoflurane in oxygen and air mixture, guided by continuous depth of anaesthesia monitoring.

Patients in the MCS arm received bolus doses of midazolam and fentanyl at the start of the procedure and prior to the first ablation delivery. Typically, this was targeted at 1–2 mg midazolam and 50 mcg fentanyl at procedure start but could be adjusted at operator discretion. Further top-up doses were provided as necessary based on degree of sedation and reported discomfort. The degree of sedation was monitored by continuous oxygen saturations and pulse rate, along with cycled blood pressure measurements. End tidal CO_2_ was also monitored using the cap35 Capnostream system (Medtronic). Sedation was also assessed by the physician when communicating with the patient throughout. Target sedation was responsiveness to voice. Major airway concerns were managed by the anaesthetist and could involve simple airway adjuncts, or conversion to endotracheal intubation if required. Continuous oxygen was delivered by nasal cannulae throughout.

At least 2 min delay was mandated between top-up doses and subsequent PFA applications to allow adequate analgesia and sedation. The operating physician kept continual contact with the patient to inform them of the procedure progress, counting down the number of PVs left to treat, and enquiring, after the first ablation delivery and intermittently throughout the remainder of the procedure, if they were finding the ablation tolerable or would prefer conversion to GA.

All ablations were performed using a pentaspline single-shot PFA catheter (FaraPulse, Boston Scientific). Access was gained to the femoral vein under ultrasound guidance, with local anaesthetic at procedure start in the MCS arm. A decapolar catheter was positioned in the coronary sinus. Transseptal puncture was performed with fluoroscopic and pressure guidance, supported by transoesophageal echocardiography in the GA arm. Following transseptal puncture, heparin was administered with target activated clotting time ≥ 300 s. PFA applications were delivered in line with previously published work [3]. Eight PFA applications were performed per vein at minimum—four in basket configuration and four in flower configuration—with additional deliveries, including ablation of the posterior wall (GA arm only), at operator discretion. PFA applications were first delivered for the left PVs followed by the right PVs. Post-ablation, protamine was administered at operator preference and closure was performed either with manual compression or a figure-of-eight suture. In the GA arm, local anaesthetic was infiltrated around the sheaths prior to withdrawal.

### Study outcome measures

The primary outcome was the need for conversion to GA in the MCS arm.

Secondary outcomes included bespoke patient experience questionnaires (included in the supplementary material) for pain, discomfort (i.e. twitching, coughing, and heart racing), and anxiety throughout the procedure. Questions on post-procedural pain and discomfort were also recorded, as was a ‘friends and family test’—i.e. would they recommend the same procedure to a friend or family member? These questionnaires were based upon previously published work from our institution [5].

Pain, discomfort, and anxiety during the procedure were rated on a scale of 0 (none) to 100 (worst imaginable), as were post-procedural groin, throat, and chest pain. ‘Discomfort’ was considered distinct from pain and instead represented symptoms such as coughing, twitching, or hiccup-like sensations during the procedure (this was explained to patients when completing the questionnaire). To assess these aspects relative to the patient’s expectations, we also applied a 5-point Likert scale ranging from ‘much worse’ (rated as 1/5 on the scale) to ‘much better’ (rated as 5/5), with a rating of 3/5 being neutral.

We also compared our data with a subset of previously published data on cryoballoon (CB) and very high-power short duration (vHPSD) radiofrequency (RF) ablation [5].

In addition, we analysed procedural metrics such as acute success, complications, lab time, fluoroscopy time, and sedative doses. Short-term follow-up outcomes were determined from review of electronic patient records.

### Statistical analysis

Continuous variables were expressed as mean ± standard deviation, or median (25th quartile – 75th quartile) depending upon the distribution, and compared using *t*-tests or non-parametric equivalents. Statistical distribution was determined with visual histogram inspection and the Shapiro–Wilk test. Categorical variables were expressed as counts and percentages and compared using Fisher’s exact test or the Kruskal–Wallis test. Statistical analysis was performed in R.

## Results

**Fig. 1:**
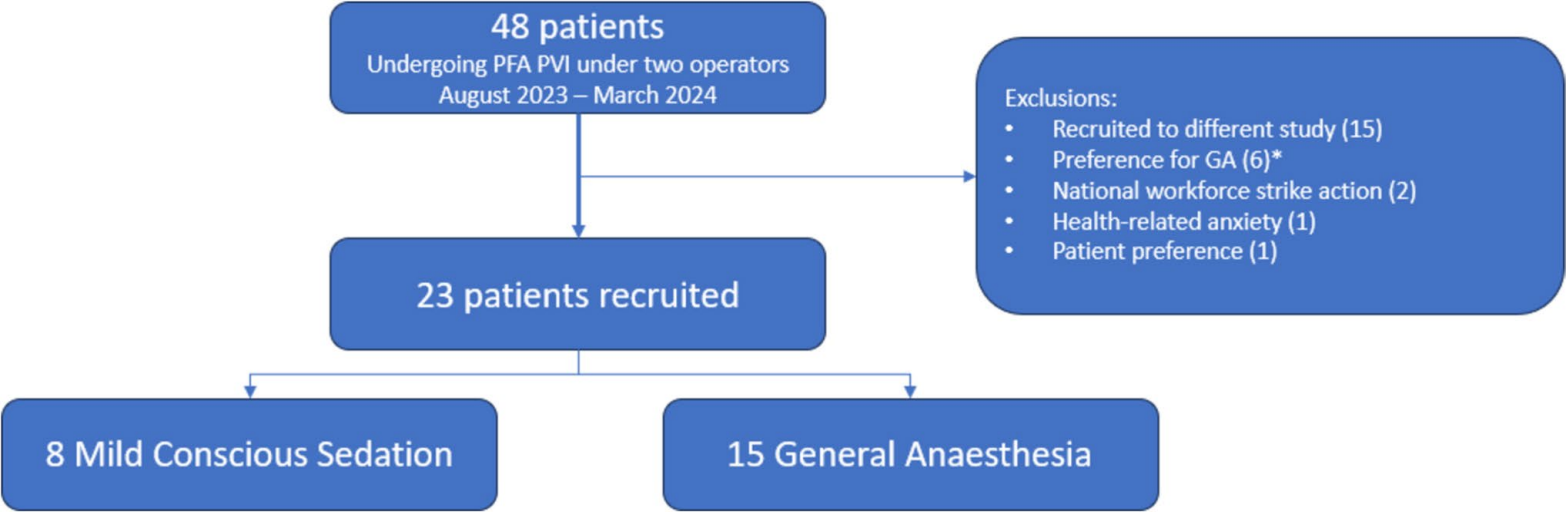
Cohort diagram. *As the GA arm was recruiting faster than the MCS arm, some patients were not approached as screening determined that they had already expressed a preference for GA only. GA, general anaesthesia; PFA, pulsed field ablation; PVI, pulmonary vein isolation

Between August 21, 2023, and March 27, 2024, 48 patients underwent PFA PVI under two electrophysiology operators (DG, VL). Of these, 23 patients were recruited for this study (8 MCS, 15 GA). The study was terminated at this stage due to difficulty in recruiting patients for the MCS arm, as the vast majority preferred to opt for GA. Additionally, as a feasibility study, our target recruitment numbers had been arbitrary, and we did not feel that our findings would significantly change if more patients were recruited based upon our experience with PFA under MCS thus far. A cohort diagram is shown in Fig. 1.

### Baseline characteristics

Demographic differences between study arms are shown in Table 1. No significant differences were detected between the groups, though the GA arm had more female patients. The main reason for opting for GA was patient preference, with only one patient having contraindications to the MCS approach (morbid obesity with obstructive sleep apnoea).

**Table 1:** Demographic differences between arms

	MCS (*n* = 8)	GA (*n* = 15)	*p*-value
Age (mean ± SD)	63 ± 9.1	61 ± 10.4	0.727
Female, *n* (%)	1 (12.5)	6 (40)	0.345
BMI (median [IQR])	27 [25–30]	30 [26–35]	0.203
AF type, *n* (%):			0.470
Paroxysmal	4 (50.0)	9 (60.0)	
Persistent	3 (37.5)	6 (40.0)	
Longstanding persistent	1 (12.5)	-	
Hypertension, *n* (%)	2 (25.0)	8 (53.3)	0.379
Diabetes, *n* (%)	-	1 (6.7)	> 0.999
Chronic kidney disease, *n* (%)	-	1 (6.7)	> 0.999
Heart failure, *n* (%)	1 (12.5)	2 (13.3)	> 0.999
Obstructive sleep apnoea, *n* (%)	-	1 (6.7)	> 0.999
CHA_2_DS_2_-VASc score (median [IQR])	1 [0–2]	1 [1–2.5]	0.291

*AF*, atrial fibrillation; *BMI*, body mass index; *GA*, general anaesthesia; *IQR*, interquartile range; *MCS*, mild conscious sedation; *SD*, standard deviation

### Procedural characteristics

Procedural characteristics in the two study arms are shown in Table 2. GA procedures were slightly longer but not to statistical significance (mean 101 min vs. 92 min; *p* = 0.199). Setup time (defined as the time from the patient entering the catheter lab until first needle puncture) was significantly quicker in the MCS arm (median 16.5 min vs. 25.0 min; *p* = 0.002) as no anaesthetic induction was required. Skin-to-skin time was similar between arms (MCS 64 min vs. GA 63 min; *p* = 0.868). Fluoroscopy time was also shorter in the MCS arm (mean 10.4 min vs. 16.9 min; *p* = 0.004), likely due to the numerically lower rate of non-PV ablation in this group (12.5% vs. 33.3%, *p* = 0.369).

**Table 2:** Procedural differences between ablation modalities

	MCS (*n* = 8)	GA (*n* = 15)	*p*-value
Total procedure time (mins)	92 ± 12.4	101 ± 17.3	0.199
Skin-to-skin time (mins)	64 ± 12.0	63 ± 15.2	0.868
Left atrial dwell time (mins)	43 ± 7.8	43 ± 12.9	0.948
Ablation time (mins)	22.5 [17.8–29.0]	21.0 [18.0–30.5]	0.974
Setup time (mins)	16.5 [14.5–18.5]	25 [20–32.5]	**0.002**
Turnaround time (mins)	11.4 ± 5.3	11.3 ± 4.4	0.984
Fluoroscopy time (mins)	10.4 ± 3.1	16.9 ± 5.1	**0.004**
Contrast dose (ml)	76 ± 42	95 ± 34	0.240
Non-PVI ablation performed, *n* (%)	1 (12.5)	5 (33.3)	0.369
Number of PFA applications	32 [32–36]	38 [33–46]	0.062
Acute procedural success (%)	100	100	> 0.999
Complications (%)	-	-	-

*GA*, general anaesthesia; *MCS*, mild conscious sedation; *PVI*, pulmonary vein isolation

Total procedure time incorporated the time from patient entering to leaving the catheter lab. Skin-to-skin time incorporated the time from first needle puncture to sheath removal. Left atrial dwell time incorporated the time from transseptal puncture to exiting the left atrium. Ablation time incorporated the time from first ablation delivery to final ablation delivery. Setup time was the time from patient entering the lab until first needle puncture. Turnaround time was the time from sheath removal to the patient exiting the catheter lab

Acute success, defined as successful isolation of all PVs, was achieved in all cases, and the number of PFA applications was similar in the MCS and GA cohorts (median 32 vs. 38; *p* = 0.062). Non-PVI ablation was additionally performed in 1 MCS patient (cavotricuspid isthmus line with a radiofrequency catheter due to atrial flutter seen during the procedure) and 5 GA patients (all of which were posterior wall ablation performed with the pentaspline PFA catheter).

No complications or adverse events were observed. No patients developed respiratory depression, apnoea, or hypotension necessitating use of reversal agents (naloxone or flumazenil) nor was airway support required in any MCS patient.

### Conversion to general anaesthesia

Of 8 patients in the MCS arm, 1 patient (12.5%) required conversion to GA due to non-tolerance of the procedure—in this case, due to pain experienced during the administration of PFA pulses, which persisted despite escalating doses of sedation and analgesia. Of the remaining patients, none requested conversion to GA.

### Sedative doses

In the MCS arm, the mean dose of midazolam per case was 5.12 ± 0.83 mg and the mean fentanyl dose was 209 ± 40 mcg. All patients additionally received intravenous paracetamol. These doses are substantially higher than those used in retrospectively compared thermal PVI procedures (mean doses: CB 1.9 mg midazolam + 131 mcg fentanyl; RF 2.0 mg midazolam + 158 mcg fentanyl) [5].

The highest MCS doses required in a single patient were 6 mg midazolam and 275 mcg fentanyl. The lowest doses were 4 mg midazolam and 150 mcg fentanyl. The absence of muscle paralytic agents (routinely given during GA) did not cause issues with patient movement.

### Patient experience

Intra-procedural pain, discomfort, and anxiety were assessed on an absolute level (0–100 scale) and relative level using a 5-point Likert scale where 1 = much worse and 5 = much better; 3 = as expected.

Pain, discomfort, and anxiety scores were generally zero in the GA cohort, suggesting anaesthesia was effective. In the MCS cohort, the median pain score was 45/100 (IQR 22.5–72.5), whilst discomfort was 0/100 (IQR 0–12.5) and anxiety was 10/100 (IQR 0–10). This may suggest reasonable tolerance outside of pain but could also reflect the amnesic effects of sedative agents.

Pain levels relative to patient expectation (by Likert analysis; lower = worse) were significantly worse with MCS (median 2.5 vs. 4; *p* = 0.027), whilst discomfort was similar between groups (MCS median 4, GA median 3; *p* = 0.466). Anxiety was better in the MCS group (median 5 vs. 4; *p* = 0.006); however, this may be affected by selection bias, as anxious patients may have preferentially opted for GA.

Post-operative groin pain was worse in the GA group (median 5/100 [0–35] vs. 0/100 [0–0]; *p* = 0.004), as was post-operative throat discomfort (median 10/100 [5–40] vs. 0/100 [0–0]; *p* = 0.001). Neither group reported significant post-procedural chest pain. The difference in groin pain may relate to the timing of local anaesthetic administration—in the MCS arm, this was given at procedure start and thus would be in full effect by the time of the questionnaire, whereas in the GA arm, it was given prior to sheath removal and potentially may not be in full effect at this timepoint.

The ‘friends and family test’, also rated on a 5-point Likert scale, was median 5 (‘definitely recommend’) in both groups; *p* = 0.869.

Notably, two patients in the MCS cohort reported no memory of any intraprocedural pain, likely as a result of the amnesic effects of midazolam. Excluding these two cases, in order to focus attention on those who recalled the procedure, the median pain score was 60/100 (Likert 2/5—‘worse than expected’). The maximum pain score, 80/100, was reported by two MCS patients, one of whom converted to GA.

Differences in patient experience are summarised in Table 3.

**Table 3:** Differences in patient experience

	MCS (*n* = 8)	GA (*n* = 15)	*p*-value
Intraprocedural pain	45 [23–73]	0 [0–0]	**0.005**
Intraprocedural discomfort	0 [0–0]	0 [0–0]	0.195
Intraprocedural anxiety	10 [0–10]	0 [0–0]	**0.014**
Post-procedural groin pain	0 [0–0]	5 [0–35]	**0.004**
Post-procedural throat pain	0 [0–0]	10 [5–40]	**0.001**
Post-procedural chest pain	0 [0–0]	0 [0–0]	0.558
Pain Likert (1–5) score	2.5 [2.0–3.5]	4.0 [4.0–5.0]	**0.027**
Discomfort Likert (1–5) score	4.0 [3.0–5.0]	3.0 [3.0–4.0]	0.466
Anxiety Likert (1–5) score	5.0 [4.0–5.0]	4.0 [3.0–4.0]	**0.006**
Friends and family test (Likert 1–5)	5.0 [5.0–5.0]	5.0 [5.0–5.0]	0.869

Scores are rated 0–100 (higher = worse). Likert scales are rated 1–5 (1 = much worse than expected, 2 = worse than expected, 3 = as expected, 4 = better than expected, and 5 = much better than expected), aside from the ‘friends & family test’ which is rated 1–5 (1 = definitely would not recommend, 2 = would not recommend, 3 = neutral, 4 = would recommend, and 5 = would definitely recommend). All values are reported as median (interquartile range)

### Short-term follow-up outcomes

Short-term follow-up was available for 16 patients, as appointments were not yet due for the remainder. Of those with follow-up (median 101 days (IQR 94–123)), 1/7 (14.3%) MCS and 3/9 (33.3%) GA patients had recurrence of AF (*p* = 0.585). Symptomatic improvement was reported in 7/7 (100%) MCS and 7/9 (77.8%) GA patients (*p* = 0.475). No patients had documented concerns about their sedation modality at follow-up review.

### Comparison vs. thermal ablation

We compared our MCS data against a prior dataset where patients at our centre underwent either CB or vHPSD RF ablation under MCS. Due to differences in study design, we did not compare discomfort, anxiety or the ‘friends and family’ test.

Median pain scores were higher with PFA as compared to thermal ablation but did not reach statistical significance (PFA 45, CB 35, vHPSD 25; *p* = 0.554). Relative pain (Likert scale, median) similarly was worse with PFA but not to significance (PFA 2.5, CB 4, RF 3; *p* = 0.337).

As with the primary analysis, a small number of patients (2/8 PFA, 4/59 CB, 5/66 RF) reported a pain score of zero, which may be influenced by significant amnesia due to midazolam. We therefore sub-analysed those patients whose pain scores were > 0. In this analysis, median pain scores were higher in all three groups and remained worse with PFA but did not reach statistical significance (PFA 60, CB 40, RF 30; *p* = 0.111). Median pain on the relative (Likert) scale was worse with PFA in these subgroups (PFA 2 [2–3], CB 4 [3–5], RF 3 [3–4]; *p* = 0.045; PFA vs. CB *p* = 0.022, PFA vs. RF *p* = 0.018, CB vs. RF *p* = 0.457).

As shown in the graphical abstract, pain described as ‘much worse’ or ‘worse’ was more frequent with PFA, whilst pain described as ‘better’ or ‘much better’ was more frequent with thermal ablation. This occurred despite the heavier sedative and analgesic doses given in the PFA cohort.

## Discussion

The introduction of PFA in many hospitals and in several countries is restricted by the lack of GA provision. As such, there is much interest in the feasibility of using alternative sedation modalities. Our study is the first to report the feasibility of PFA with the FaraPulse catheter performed under MCS. Our primary findings are:

1. PFA with the FaraPulse catheter under MCS is feasible, though this approach may be challenging for reasons discussed below.
2. There are benefits to PFA under MCS in terms of avoiding anaesthetic side effects and better post-procedural experience, but these may be offset by discomfort caused by the procedure.
3. Most patients preferred to have their procedure performed under GA a priori once they had engaged in an open and transparent discussion with the caregiver regarding the pros and cons of MCS vs. GA.
4. Intraprocedural pain perception with PFA under MCS was worse than a retrospective group undergoing CB or RF ablation, despite much higher sedative and analgesic doses.

### When, if ever, should PFA be performed under conscious sedation?

Our study shows the feasibility of PFA under MCS. This raises the question of whether this approach should see widespread adoption. We would suggest not, for the reasons described below.

First, patient-reported pain scores were high, even allowing for the amnesic effects of intraprocedural benzodiazepines. Whilst it may be argued that some discomfort is to be expected for an interventional procedure such as PVI, this must be offset by the benefits of the procedure itself. In the absence of conclusive advantages of PFA vs. thermal ablation [1, 2], it is difficult to justify the higher pain perception with PFA, especially given the added cost of PFA equipment [7] in a procedure primarily intended to provide symptomatic improvement. Of note, in routine practice, thermal ablation tends to cause persisting discomfort, which may take a few weeks to resolve post-procedure. This is avoided with PFA, where the discomfort wears off almost immediately. This is an advantage under GA; however, we did not feel that the level of discomfort experienced under MCS justifies adopting this approach.

Second, high bolus sedative doses were required in the MCS arm (with total doses as high as 6 mg midazolam and 275 mcg fentanyl). Whilst there are generally no defined ‘upper limits’ to these medications, these doses are substantially higher than those used in routine electrophysiology practice and were much higher than those used in comparative thermal PVI procedures (mean doses CB 1.9 ± 1.6 mg midazolam and 131 ± 60 mcg fentanyl; RF 2.0 ± 2.2 mg midazolam and 158 ± 49 mcg fentanyl) [5]. This may increase the risk of airway compromise, though our study lacks statistical power to capture uncommon complications. Our findings support a recent study showing that, even with deep sedation, sedative requirements for PFA may be higher than for thermal ablation modalities [8]. Furthermore, though we did not attempt posterior wall ablation in the MCS group, our findings suggest that tolerance may be poorer, and even higher sedative doses would be required.

Based upon our experience, therefore, we would suggest that PFA under MCS be reserved for unusual circumstances. For example, prior to this study, we had performed one case of PFA under MCS on a patient planned for RF ablation where the RF generator malfunctioned following the transseptal puncture. In this case, the PFA equipment was already set up from a prior GA case—the patient was offered either re-listing or an attempt at PFA under MCS; they opted for, and tolerated, the latter reasonably well.

Future studies could be considered utilising alternative sedation techniques, different PFA waveforms—or different PFA catheters—in order to determine the optimal approach. If PFA could be optimised to minimise skeletal muscle capture—which appears to be responsible for the majority of discomfort experienced—PFA under MCS may become a viable strategy in the future.

### How can PFA uptake be improved in health systems where GA is limited?

As discussed earlier, it is routine practice in many centres to perform ablation under deep sedation, using a propofol infusion, without anaesthetic support. This approach has a demonstrable safety record [9–16] since the early 2010s. Nonetheless, this practice continues to be disallowed in the UK and several other countries around the world.

Bolus dosing of midazolam and fentanyl may carry inherent risk. Patients may respond differently to different doses, and airway risk may be unpredictable. Although these drugs have reversal agents available, administration of a reversal agent may make continuing the ablation procedure unfeasible. In contrast, propofol given as an infusion has been shown to be highly predictable, easily titratable, wears off quickly when stopped, and provides superior levels of sedation, thereby improving patient experience [12, 17].

Although propofol-based sedation would likely be superior to MCS for thermal ablation, these modalities in the past did not provide any specific benefit—i.e. clinical outcomes were similar whether MCS or GA was utilised. Now, however, PFA has entered the arena. PFA is theoretically safer due to cardioselectivity [18] and improves procedure times and throughput [2]. As tolerability of PFA under MCS is suboptimal in our experience from this study, it may be time for a renewed call for the use of propofol-based deep sedation. This would, of course, necessitate the development of careful protocols and appropriate training, developed in conjunction with anaesthetic specialists, ensuring appropriate patient selection—for example, patients with elevated BMI [9] and/or obstructive sleep apnoea [19] may be unsuitable due to airway risk—and intraprocedural monitoring.

GA will continue to have a role in invasive cardiac electrophysiology, for those patients who prefer this approach, experience pain despite even deep sedation [20], and for cases where catheter stability may be particularly important. This may factor into newer PFA catheters with integration into electroanatomic mapping systems—map shifts due to movement caused by ablation delivery may necessitate intraprocedural paralytics, although this has not proven problematic in studies performed thus far [12, 13].

## Limitations

This was a single-centre study in the UK; our findings may not be generalisable to other centres, healthcare systems, operators, or other PFA systems. Our study was not randomised, so unmeasured bias is likely to be present—however, the purpose was simply to analyse the feasibility of this approach in optimally selected patients. Our numbers were low so underpowering is likely. Although we used a combination of midazolam and fentanyl to facilitate MCS, alternative strategies may also be viable (e.g. remifentanil infusion and/or patient-controlled analgesia)—our study should not be generalised to other MCS approaches. Our study questionnaires were bespoke, without external validity testing; however, they were primarily intended to capture the degree of patient tolerance in a manner comparable to previously published work, thereby facilitating comparison. However, the thermal comparison study was designed differently from the present study, and this may result in artefactual differences between the modalities.

## Conclusion

PFA performed under MCS is viable in selected patients, but pain and tolerance may be suboptimal, and high sedative doses are required. This underscores the need for availability of GA or deep sedation in centres with access to PFA.

## Supplementary Information

Below is the link to the electronic supplementary material.

Supplementary file1 (DOCX 216 KB)

## Data Availability

For confidentiality of participants, data cannot be made public but may be made available anonymously upon reasonable request to the corresponding author.
