# Supplementary material for: Feasibility of pulsed field ablation for atrial fibrillation under mild conscious sedation
Source: J Interv Card Electrophysiol. 2024 Dec 2;68(7):1429–36. doi: 10.1007/s10840-024-01961-1 (PMC12436502; doi:10.1007/s10840-024-01961-1)
Supplement: Supplementary file 1 — Supplementary file1 (DOCX 216 KB) [file 10840_2024_1961_MOESM1_ESM.docx]

**Patient Experience**

We would like you to rate your experience of pain and anxiety during your procedure on a scale from 0 to 100. 0 represents no pain or anxiety at all, whilst 100 represents the worst pain or anxiety you can imagine.


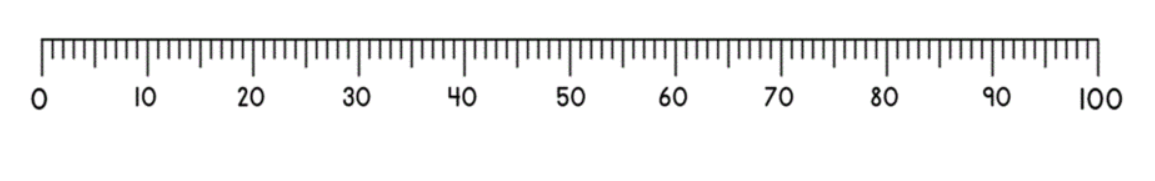


No pain or anxiety at all

Worst pain or anxiety imaginable

| Please rate, on a scale of 0 to 100, how much pain you felt overall during your procedure |  |
| --- | --- |
|  |  |
| Please rate, on a scale of 0 to 100, how much discomfort* you felt overall during your procedure |  |
|  |  |
| Please rate, on a scale of 0 to 100, how much anxiety you felt overall during your procedure |  |

**By “discomfort”, we mean uncomfortable sensations such as muscle twitching / jerking, hiccups, coughing, etc.*

Now that the procedure is over, please could you rate how much pain you are currently experiencing in the following places (0 to 100 scale):

| Groin |  | Throat |  | Chest |
| --- | --- | --- | --- | --- |
|  |  |  |  |  |

**Patient Experience**

How did your experience compare to what you expected? Please **tick one box in each row** in the table below which best describes your experience.

|  | Much worse than I expected | Worse than I expected | About the same as I expected | Better than I expected | Much better than I expected |
| --- | --- | --- | --- | --- | --- |
| PAIN | 🞎 | 🞎 | 🞎 | 🞎 | 🞎 |
| DISCOMFORT | 🞎 | 🞎 | 🞎 | 🞎 | 🞎 |
| ANXIETY | 🞎 | 🞎 | 🞎 | 🞎 | 🞎 |

Based on your experience, would you recommend this procedure to a friend or family member, if they had the same condition? **Tick one box.**

|  | Definitely not | Probably not | Neutral | Probably | Definitely |
| --- | --- | --- | --- | --- | --- |
|  | 🞎 | 🞎 | 🞎 | 🞎 | 🞎 |

This concludes the patient experience questionnaire. If you have any additional comments, please feel free to write them below. Otherwise, please hand this form back to your doctor or nurse.
